# Frequency-Dependent Fatigue Properties of Additively Manufactured PLA

**DOI:** 10.3390/polym16152147

**Published:** 2024-07-29

**Authors:** Martin Česnik, Janko Slavič

**Affiliations:** Faculty of Mechanical Engineering, University of Ljubljana, Aškerčeva 6, 1000 Ljubljana, Slovenia; martin.cesnik@fs.uni-lj.si

**Keywords:** fatigue properties, frequency dependence, vibration-fatigue test, high-frequency dynamic response

## Abstract

Vibration-fatigue failure occurs when a structure is dynamically excited within its natural frequency range. Unlike metals, which have constant fatigue parameters, polymers can exhibit frequency-dependent fatigue parameters, significantly affecting the vibration resilience of 3D-printed polymer structures. This manuscript presents a study utilizing a novel vibration-fatigue testing methodology to characterize the frequency dependence of polymer material fatigue parameters under constant temperature conditions. In this investigation, 3D-printed PLA samples with frequency-tunable geometry were experimentally tested on an electro-dynamical shaker with a random vibration profile. Using the validated numerical models, the estimation of vibration-fatigue life was obtained and compared to the experimental results. Performing the numerical minimization of estimated and actual fatigue lives, the frequency-dependent fatigue parameters were assessed. In particular, the results indicate that the tested samples exhibit varying fatigue parameters within the loading frequency range of 250–750 Hz. Specifically, as the loading frequency increases, the fatigue exponent increases and fatigue strength decreases. These findings confirm the frequency dependence of fatigue parameters for 3D-printed polymer structures, underscoring the necessity of experimental characterization to reliably estimate the vibration-fatigue life of 3D-printed polymer structures. The utilization of the introduced approach therefore enhances the vibration resilience of the 3D-printed polymer mechanical component.

## 1. Introduction

Fused Filament Fabrication (FFF) [1] is the most popular additive-manufacturing (AM) technology, primarily using thermoplastics and researched for diverse applications, ranging from mechanical components to printed sensors [2] and metamaterials [3]. Its use of biodegradable materials such as polylactic acid (PLA) makes it a promising alternative to conventional injection molding. Additionally, the advanced polymer materials (PEEK and PEI) [4] with short and long fibers can completely replace metals in mechanical components. However, ensuring the reliability of FFF components under dynamic loads requires comprehensive experimental characterization, as their mechanical properties depend heavily on processing parameters (e.g., temperature, nozzle diameter, and infill pattern) [5,6,7] and environmental conditions (e.g., temperature and humidity) [8].

**Figure 1:**
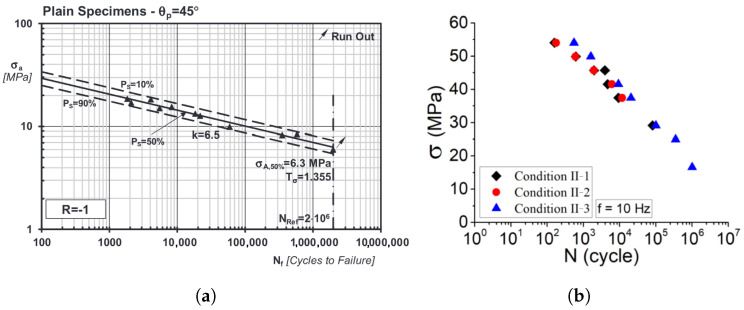
Wöhler curves indicating linearity in log–log scale for (**a**) plain PLA [17] and (**b**) graphene-PLA [18].

Most studies on FFF components focus on static tensile and flexural tests, but interest in their fatigue properties is growing, as noted by Rouf et al. [8]. Early fatigue studies include work by Afrose et al. [9], Jerez-Mesa et al. [10], and Gomes-Gras et al. [11], examining the low-cycle fatigue of FFF PLA. Ezeh et al. [12,13] explored high-cycle fatigue, load ratios, raster orientation, and notch effects. Mayén et al. [14] studied raster angle and heat treatment on FFF PLA fatigue life. Research also extends to other materials like PEEK, ABS, wood-induced PLA, PA6, and carbon-fiber-reinforced PA6, as shown by Rendas et al. [10], He et al. [11], Ziemian et al. [12], Travieso-Rodrigues et al. [13], Terekhinna et al. [14], and Li et al. [15], respectively. Additionally, AM metal components [16] also exhibit anisotropic fatigue parameters, which can be manifested in vibration-fatigue tests. On the other hand, the experimental results of fatigue tests show that the additively manufactured polymer materials exhibit linear fatigue lives in the log–log scale and in agreement with the Wöhler curve and Basquin’s equation. An exemplary of Wöhler curves of additively manufactured polymer, namely plain PLA and PLA-graphene, are shown in Figure 1a [17] and Figure 1b [18], respectively.

The fatigue tests in the aforementioned studies were performed at low loading frequencies (4–30 Hz) to prevent excessive heat generation within the sample. As reported by Shanmugam et al. [19] and Crawford and Martin [20], accelerating the fatigue tests by increasing the loading frequency leads to ductile fatigue failure and a significantly shorter fatigue life. Similarly, Kuleyin et al. [21] observed high differences in the fatigue lives of PMMA/ABS blends for loading frequencies between 5 Hz to 25 Hz, which they attributed to the temperature increase up to 31 °C. Considering a high frequency dependence of polymer’s mechanical properties, the obtained Wöhler curves can be reliably utilized only for the evaluation of the dynamic loads within the frequency range of performed fatigue test. Due to the high frequency dependence of polymer mechanical properties, the resulting Wöhler curves are only reliable for evaluating dynamic loads within the frequency range used in the fatigue tests. Recently, additively manufactured structures are becoming more prevalent in applications involving dynamic loading and external vibrations. In this light, Nguyen et al. [22] investigated the influence of adhesion type on the natural frequency and damping ratio of additively manufactured ABS specimens. Xue et al. [23] focused on the influence of processing parameters on the natural frequencies, mode shapes, and damping ratios of FFF-manufactured PLA components, while Medel et al. [24] observed the repeatability of the additively manufactured structure for a defined set of processing parameters. The dynamic response of the 3D-printed structure can also be utilized to characterize the structure’s orthotropic material constants [25].

Moreover, when the external dynamic loads lie within the frequency range of the structure’s natural frequencies, failure due to vibration fatigue [26] can occur. The first research on the vibration fatigue of a 3D-printed polymer structure was reported by Palmieri et al. [27]. However, the increasing popularity of 3D-printed sensors [28,29] and actuators [30] indicate the rising need to reliably address the vibration fatigue of 3D-printed polymer structures. The analysis of the structure’s fatigue life requires the material’s fatigue properties, namely fatigue exponent and fatigue strength, which can only be obtained, with existing methodologies, by a low-frequency fatigue test. This can introduce a high error in the fatigue life estimation, since, currently, there is no existing knowledge of the loading frequency influence on the polymer’s fatigue exponent and fatigue strength. Recently, a new vibration-based methodology was introduced [31], which utilizes the dynamic response of frequency-tunable specimens with a natural frequency range from 250 Hz to 750 Hz. This manuscript presents the research on frequency-dependent fatigue properties of additively manufactured PLA specimens, which can be experimentally investigated with the vibration fatigue of frequency-tunable samples.

This manuscript is organized as follows. Section 2 presents an overview of the vibration-based fatigue-testing methodology together with the sample’s geometry, material, and processing parameters. In Section 3, the experimental results are presented and analyzed in detail. The discussion on the obtained results is given in Section 4 together with the conclusions.

## 2. Methods and Materials

This manuscript investigates the frequency dependency of 3D-printed polymer structures using a vibration-based fatigue testing method, as detailed in [31] and outlined in Section 2.1. Section 2.2 presents a detailed description of the frequency-tunable samples used to characterize the frequency dependence of the fatigue parameters.

### 2.1. Methods

**Figure 2:**
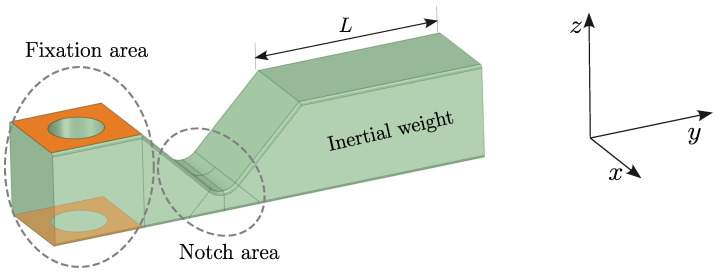
Geometry of sample for vibration-based fatigue testing.

The vibration-based fatigue test method adopted an innovative sample design, which consisted of a fixation area, a notch area, and an inertial weight. The sample geometry and its features are presented in Figure 2. The proposed sample geometry has 3 important characteristics: it is frequency-tunable, it can be manufactured by FDM in three spatial axes, and it exhibits a near-uniaxial stress state in the fatigue zone at the notch. More details on the sample’s geometry are given in [31].

**Figure 3:**
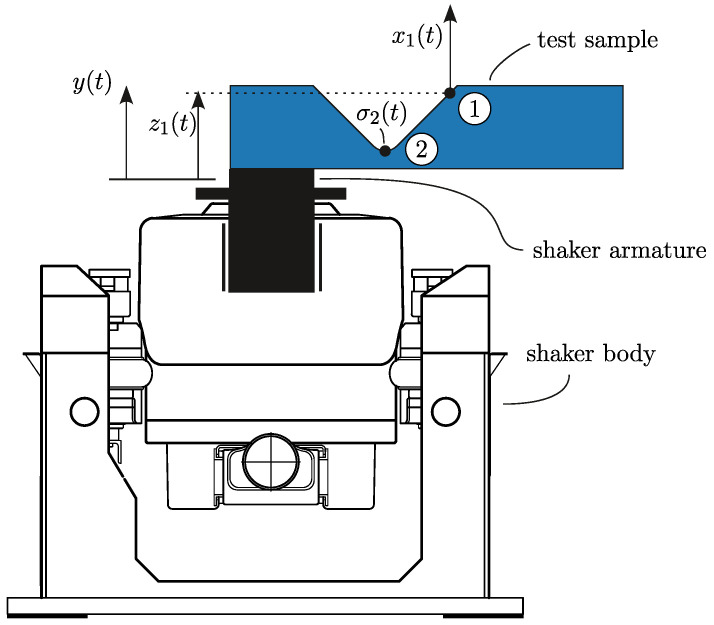
Concept of vibration-based fatigue test (for clarity, the sample is oversized): annotated 1 denotes the location of response measurement, annotated 2 denotes the fatigue zone.

By mounting the test sample on the electro-dynamic shaker, as illustrated in Figure 3, the sample can be treated as a dynamic system and can be excited within the frequency range of 5–2000 Hz. Within the vibration-based fatigue test [31] the loading is a random-signal acceleration with a constant-level power spectral density (PSD). Due to the random nature of the excitation displacement y(t) and acceleration $\ddot{y}\left(t\right)$ at the shaker armature and the stress response in the fatigue zone $\sigma_{2}\left(t\right)$, the analysis was performed in the frequency, rather than in the time, domain. The relationship between excitation y(ω) and stress response $\sigma_{2}\left(\omega \right)$ and its relation to vibration-fatigue life is explained next.

Assuming that a stress load $\sigma_{2}$ is proportional to the relative displacement $z_{1}=x_{1}-y$, the stress transmissibility of the sample is

(1) $$H_{\sigma \ddot{y},2}\left(\omega \right)=\sum_{r=1}^{N}\frac{\Gamma_{r}{\;}_{\sigma }\phi_{r,2}}{\omega_{r}^{2}-\omega^{2}+i\;\eta_{r}\;\omega_{r}^{2}},$$

where $\Gamma_{r}$ denotes the *r*-th mode participation factor [31,32], ${\;}_{\sigma }\phi_{r}$ denotes the *r*-th stress mode shape, and $\omega_{r}$ and $\eta_{r}$ are the *r*-th natural frequency and damping ratio [33], respectively. $\omega_{1}=2\pi \cdot f_{1}$ describes the lowest frequency with which the dynamic system naturally oscillates when displaced from the equilibrium state, and $\eta_{1}$ defines the attenuation rate of the natural oscillation at the 1st natural frequency. While the $\omega_{1}$ can be obtained analytically by solving the eigenvalue problem or experimentally with one of the methods of modal analysis [33], the damping ratio $\eta_{1}$ can only be obtained from experimental data.

In Equation (1), subscript 2 denotes the fatigue zone location, as enumerated in Figure 3. With known stress transmissibility $H_{\sigma \ddot{y},2}\left(\omega \right)$, the stress response can be defined as

(2) $$\sigma_{2}\left(\omega \right)=H_{\sigma \ddot{y},2}\left(\omega \right)\cdot \ddot{y}\left(\omega \right)$$

When the dynamic system is kinematically excited with stationary random-signal acceleration with PSD $G_{\ddot{y}\ddot{y}}\left(\omega \right)$ [34], the stress response at the fatigue zone can also be obtained in terms of PSD as $G_{\sigma \sigma ,2}\left(\omega \right)$ [26]:

(3) $$G_{\sigma \sigma ,2}\left(\omega \right)={\left|H_{\sigma \ddot{y},2}\left(\omega \right)\right|}^{2}G_{\ddot{y}\ddot{y}}\left(\omega \right).$$

Stress PSD $G_{\sigma \sigma ,2}\left(\omega \right)$ and the material fatigue parameters provide sufficient information to obtain a fatigue-life estimation by utilizing spectral damage counting methods. Material fatigue parameters, namely fatigue strength exponent *b* and fatigue strength coefficient $S_{f}$, describe Wöhler’s curve with Basquin’s equation as $\sigma =S_{f}N^{b}$, where σ denotes the stress-cycle amplitude and *N* denotes the number of full cycles. Physically, *b* describes the rate at which the fatigue strength decreases with an increasing number of cycles, and $S_{f}$ is the theoretical stress at which the material would fail after a single cycle. The preceding studies [35,36,37] show the decrease of $S_{f}$ and changes of *b* at higher loading frequencies as a direct consequence of the heat generation and increased temperature in the fatigue zone.

When a dynamic structure is excited with a stationary random signal in the frequency range of a single natural frequency, the stress response can be regarded as a stationary narrow-band signal [38]. In such a case, the amplitudes of the load cycles follow a Rayleigh distribution [39], yielding an analytically deductible relation for the damage intensity (i.e., damage over time unit), known as the narrowband method [39]:

(4) $$d^{\mathrm{NB}}=\frac{{\left(\sqrt{2\;m_{0}}\right)}^{k}}{2\;\pi \;C}\sqrt{\frac{m_{2}}{m_{0}}}\;\Gamma \left(1+\frac{k}{2}\right),$$

where k=−1/b, $C=S_{f}^{-1/b}$, Γ is the Gamma function, and $m_{0}$ and $m_{2}$ are the zeroth and second moment of stress response PSD, following [34]:

(5) $$m_{i}=\int_{0}^{+\infty }\omega^{i}\;G_{\sigma \sigma }\left(\omega \right)\;d\omega .$$

when the stress response does not resemble the narrowband signal, but is still of stationary nature, a range of alternative spectral methods are available [26].

**Figure 4:**
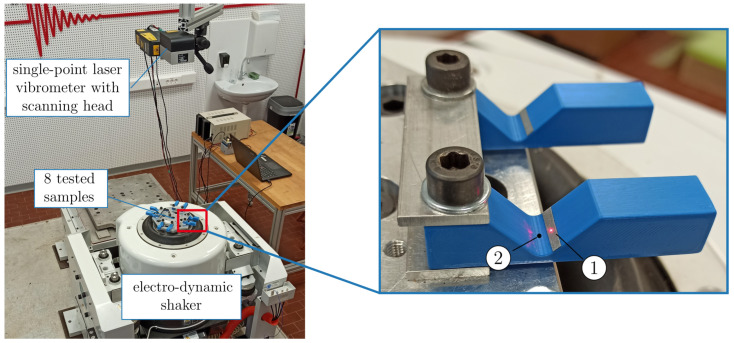
Experimental setup of vibration-based fatigue test with a detailed view of the two samples.

The experimental setup of the vibration-based fatigue test is depicted in Figure 4. Evidently, the vibration-fatigue test can be performed simultaneously on multiple samples. Furthermore, since the excitation is an acceleration with a flat-shaped PSD profile, the single experiment setup can consist of samples with different inertial weight length *L*.

A detailed view in Figure 4 shows that no strain gage was installed on the individual test sample. Instead, an indirect stress measurement was performed by measuring the velocity response on the position 1 with a laser vibrometer. With the measured transmissibility of each sample, a valid stress response PSD was obtained using a validated numerical model. More detail on the indirect stress measurement and its implementation in the vibration-fatigue test is given in [31]. Additionally, a scanning head was mounted on the laser vibrometer, which enabled the monitoring of transmissibility on multiple samples during the vibration-fatigue test. The transmissibility measurements during the fatigue test provided information on the changes of natural frequency and damping ratio.

### 2.2. Materials

In a vibration-based fatigue test, the loading frequency corresponds to the sample’s natural frequency. Hence, to investigate the frequency dependence of fatigue parameters for 3D-printed polymers, the natural frequencies of the tested samples must cover a broad frequency range. With the proposed sample geometry (Figure 2), this can be easily achieved with variations of the inertial weight length *L*. The preceding study [31], which introduced the vibration-based fatigue test, also presented the set of samples, produced with constant processing parameters and different weight lengths *L*. This resulted in a range of samples with natural frequencies from 250 Hz to 700 Hz. An overview of these samples, used in this manuscript for investigating frequency-dependent fatigue parameters, is given next; an interested reader can find more details on the samples in [31].

**Figure 5:**
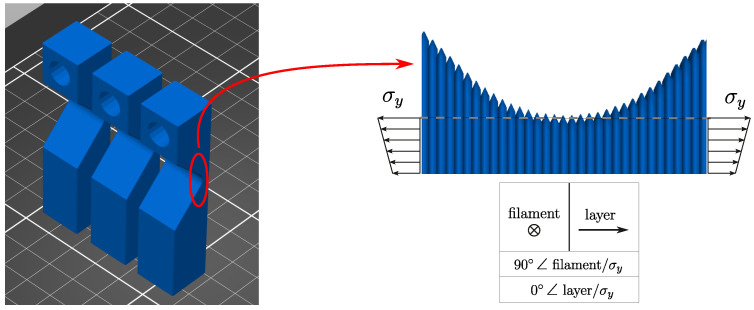
Sample’s position on the 3D-printing plate and the filament and layer orientation in the sample’s fatigue zone.

All samples tested were printed in the *y*-direction, yielding orthogonality between filaments and normal stress $\sigma_{y}$ and parallelism between layer-normal and normal stress $\sigma_{y}$, as shown in Figure 5. For 3D-printing, a Prusa i3 MK3S+ printer (Prague, Czech Republic) was used. Relevant material properties and processing parameters are specified in Table 1.

In total, 30 samples were manufactured with weight lengths *L* raging from 9 mm to 30 mm with 3 mm intervals. According to the preliminary experimental analysis, the two levels of acceleration load PSD $G_{\ddot{y}\ddot{y}}\left(\omega \right)$ were used, namely 0.1 g^2^/Hz and 0.15 g^2^/Hz. The quantities of samples with particular weight length *L* and tested with a particular $G_{\ddot{y}\ddot{y}}\left(\omega \right)$ level are listed in Table 2. The frequency range of $G_{\ddot{y}\ddot{y}}\left(\omega \right)$ was 200–800 Hz, which covered all possible values of the sample’s natural frequencies.

**Figure 6:**
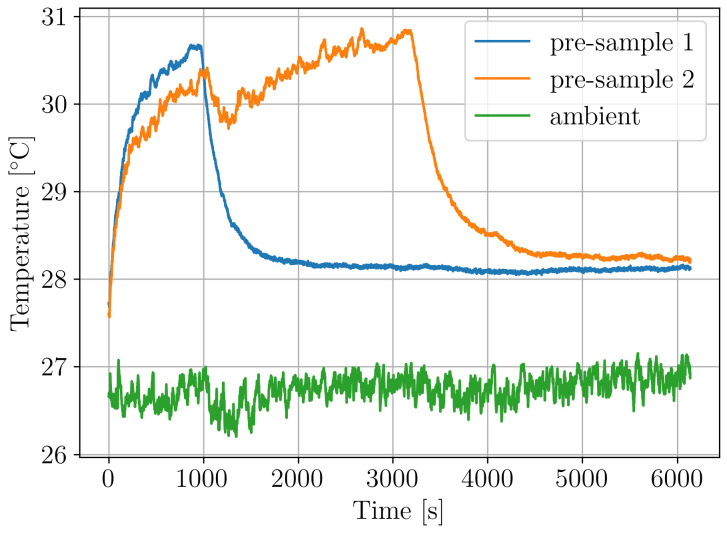
Temperature increase during vibration-fatigue test.

Before conducting the actual vibration-fatigue tests, the temperature in the fatigue zone was measured using a thermocouple on two pre-samples, excited with a $G_{\ddot{y}\ddot{y}}\left(\omega \right)$ level of 0.2 g^2^/Hz. The measured temperatures in the pre-sample’s fatigue zone are presented together with an ambient temperature in Figure 6. Evidently, the temperature increase due to stress load during fatigue test is 3 °C.

## 3. Results

### 3.1. Experimental Results

**Figure 7:**
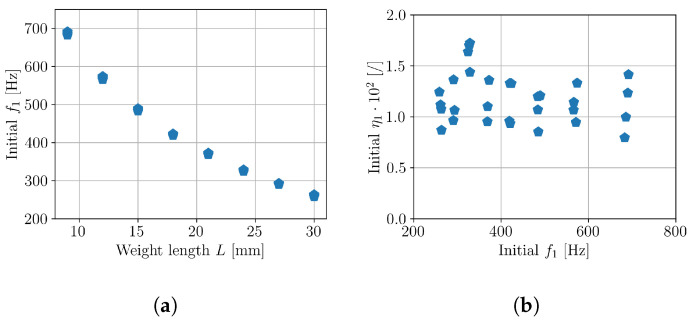
Initial modal parameters of tested samples; (**a**) natural frequencies $f_{1}$; (**b**) damping ratios $\eta_{1}$.

Initially, the actual natural frequencies and damping ratios (Equation (1)) were characterized to confirm that the manufactured samples corresponded to the expected natural frequency range of 200–750 Hz and that the modal parameters ($f_{1},\eta_{1}$) did not deviate between the manufactured samples with the same geometry. The identification of $f_{1}$ and $\eta_{1}$ was performed using the Least-Squares Complex Frequency (LSCF) method [40,41] on the initial measured transmissibility of each sample. Figure 7 shows the obtained sets of $f_{1}$ and $\eta_{1}$, confirming a good coherence between the samples.

**Figure 8:**
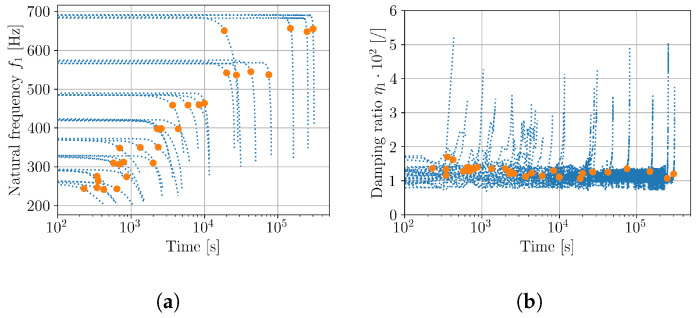
Initial modal parameters of tested samples; (**a**) natural frequencies $f_{1}$, (**b**) damping ratios $\eta_{1}$. Orange markers denote the end of the samples’ fatigue life.

Next, the samples were fatigue-tested on an electro-dynamic shaker with random signals, defined in Section 2.2 and in Table 2. As shown in Figure 4, the response was monitored with a laser vibrometer throughout the fatigue test. From the measured transmissibilities, the natural frequencies and damping ratios were extracted with a similar approach as for initial modal parameter assessment (Figure 7). Results in Figure 8a indicate the natural frequencies of samples and damping ratios. In vibration fatigue, significant changes in natural frequencies and damping ratios are regularly observed before the complete breakage of the test sample occurs and can be used as an estimator for failure declaration [42,43]. For the PLA samples tested, a threshold of a 5% frequency drop was adopted for declaring the fatigue failure of a tested sample. Orange markers in Figure 8 indicate when the 5% drop of natural frequency was exceeded. Accordingly, it is reasonable to assume that, within the fatigue life period, the damping and natural frequency changes did not have significant impact on the dynamic response (Equation (1)) of the sample.

### 3.2. Vibration-Fatigue Lives

**Figure 9:**
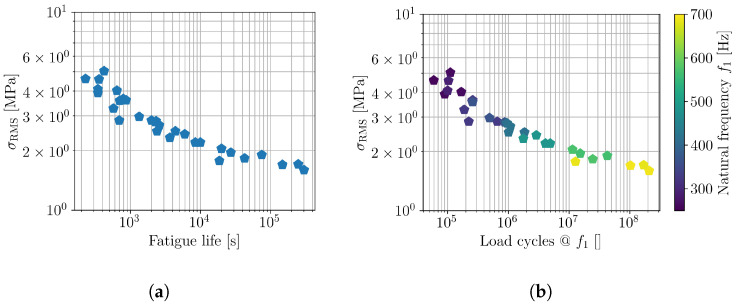
Experimental results; (**a**) fatigue lives and (**b**) number of cycles until failure.

Direct results of the vibration-fatigue test provide the fatigue life of each sample in relation to the $G_{\ddot{y}\ddot{y}}\left(\omega \right)$ level. Since the geometry of the samples varied according to Table 2 and the stress response also depended on the samples’ stress transmissibility (Equation (1)), it is more feasible to observe the results in in terms of the stress response, obtained with valid numerical model [31]. An initial estimator for stress response is the stress root-mean-square (RMS) value, also obtained as a first spectral moment [34] of $G_{\sigma \sigma }\left(\omega \right)$. The vibration-fatigue test results in Figure 9a present the actual time-to-failure, and Figure 9b presents the number of cycles until failure. One can observe a good coherence between the obtained fatigue lives and a wide range of obtained cycles to failure (from $6\times 10^{4}$ cycles to $2\times 10^{8}$ cycles).

According to the vibration-fatigue theory [26] and spectral counting methods (narrowband, Dirlik) in particular, the relation between $\sigma_{\mathrm{RMS}}$ and load cycles to failure should be a linear function in log–log scale, similar to the classic Wöhler curve. However, the results shown in Figure 9 exhibit clear deviations from the linear dependency, as observed in preceding studies and exemplified in Figure 1. Consequently, Figure 8b also includes the information of the test samples’ natural frequency. Clearly, the inclination of the $\sigma_{\mathrm{RMS}}$ vs. load-cycles curve depends on the samples’ natural frequency, which coincides with the loading frequency. To this end, the potential frequency dependence of fatigue parameters is further analyzed.

### 3.3. Frequency-Dependent Fatigue Parameters

Although Figure 9 summarizes the experimental results, it cannot be directly used to obtain the sought fatigue parameters *b* and $S_{f}$. Instead, a numerical procedure using individually validated numerical models was adopted, as described next.

Within the experimental work, the tested samples differed in terms of general geometry (length *L*) and in terms of modal parameters ($f_{1}$ and $\eta_{1}$). Accordingly, the stress PSD $G_{\sigma \sigma }\left(\omega \right)$ had to be obtained individually for every specimen. With a known $G_{\sigma \sigma }\left(\omega \right)$, a damage estimation can be obtained using the Dirlik spectral method [44], where fatigue parameters *b* and $S_{f}$ present the input parameters. However, in the case of present study, the fatigue parameters were unknown variables, while the damage estimation was a known input according to the experimental results of the vibration-fatigue test. Parameters *b* and $S_{f}$ could not be deduced analytically by mathematical manipulation; therefore, a numerical approach with a cost function Q(b,Sf) minimization was adopted. The cost function *Q* is defined as [31]

(6) $$Q\left(b,S_{f}\right)=\frac{1}{N}\;\sum_{i=1}^{N}(\log_{10}{\left(T_{\mathrm{act},i})-\log_{10}\left(T_{\mathrm{est},i}\left(b,S_{f}\right)\right)\right)}^{2}.$$

**Figure 10:**
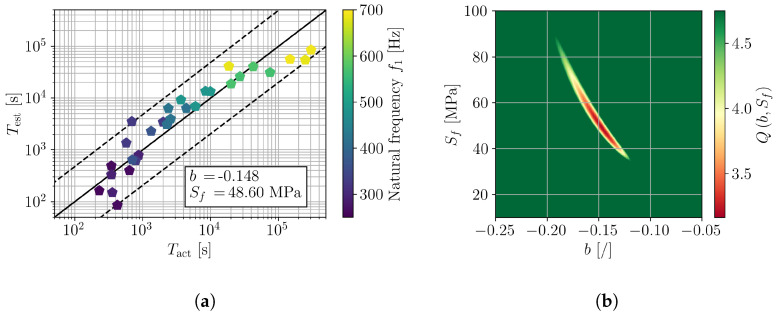
Assessment of *b* and $S_{f}$ on a complete sample set with $f_{1}$ in [250, 700] Hz; (**a**) comparison of actual and estimated fatigue lives and (**b**) error function $Q(b,S_{f})$.

Considering, first, the complete range of experimental results (Figure 9), the minimization of $Q(b,S_{f})$ yields the fatigue parameters b=−0.148 and $S_{f}=48.6$ MPa, which can also be observed in the error plot in Figure 10b. The comparison of actual and estimated fatigue lives of the samples for such a pair of fatigue parameters is shown in Figure 10a, where one can clearly observe that the actual fatigue lives cannot be adequately described with a single pair of ($b,S_{f}$). Considering the results of preceding studies on the fatigue of polymers (e.g., [17,18,31]) the observed deflection from the linear relation between $T_{\mathrm{act}}$ and $T_{\mathrm{est}}$ can only be attributed to the frequency-dependent fatigue parameters.

**Figure 11:**
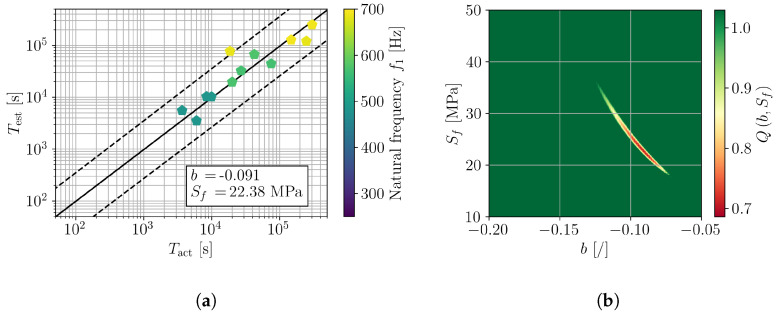
Assessment of *b* and $S_{f}$ on samples with $f_{1}$ in [450, 700] Hz; (**a**) comparison of actual and estimated fatigue lives and (**b**) error function $Q(b,S_{f})$.

**Figure 12:**
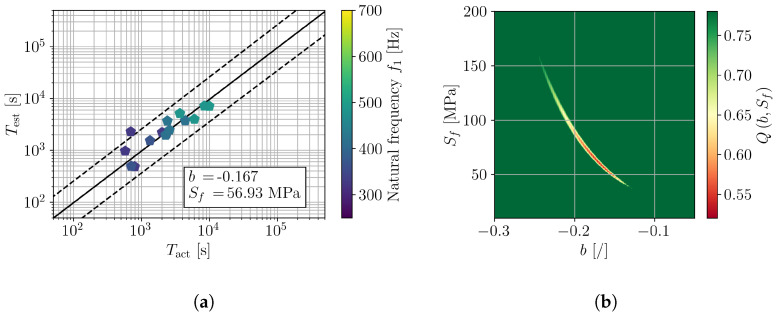
Assessment of *b* and $S_{f}$ on samples with $f_{1}$ in [300, 500] Hz; (**a**) comparison of actual and estimated fatigue lives and (**b**) error function $Q(b,S_{f})$.

**Figure 13:**
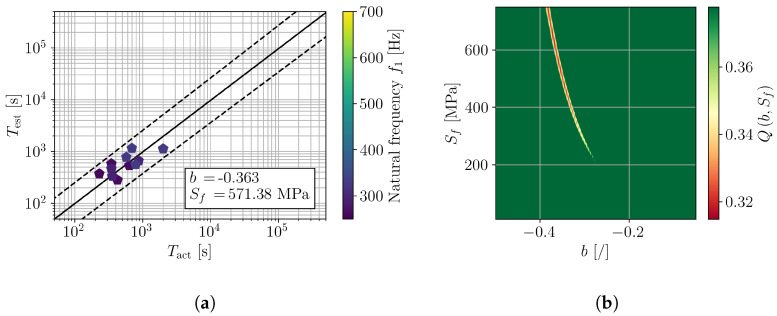
Assessment of *b* and $S_{f}$ on samples with $f_{1}$ in [250, 350] Hz; (**a**) comparison of actual and estimated fatigue lives and (**b**) error function $Q(b,S_{f})$.

**Figure 14:**
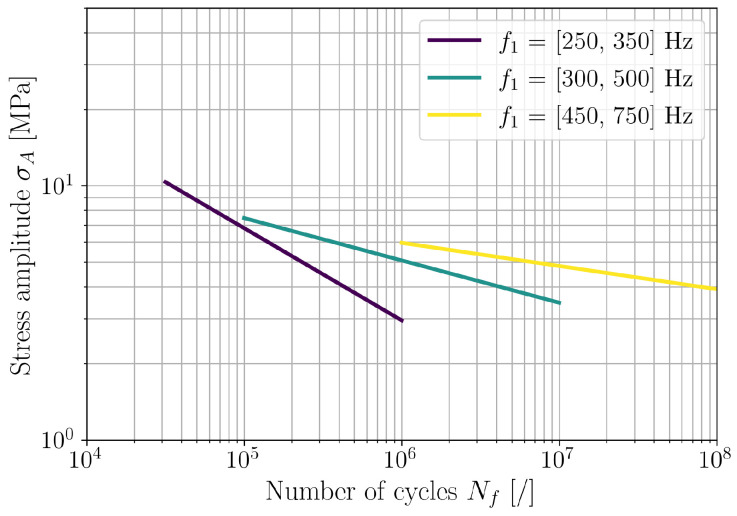
Frequency-dependent SN curves of 3D-printed PLA.

For a detailed investigation of the frequency dependence of $\left(b,S_{f}\right)$, the samples were divided into groups according to their natural frequency (Figure 7). In particular, three ranges of natural frequency were defined, e.g., [250, 350] Hz, [300, 500] Hz, and [450, 700] Hz. The results are presented in Figure 11, Figure 12 and Figure 13 in a similar manner as for all tested samples in Figure 10. The obtained fatigue parameters are also listed in Table 3. The SN curves, derived according to the obtained frequency-dependent fatigue parameters, are presented in Figure 14. The cycle range, where the specific curve is shown, is defined by the minimum and maximum load cycles of the samples within the given range of natural frequencies; see Figure 9b.

## 4. Discussion and Conclusions

The presented results confirm an importance of loading frequency on the fatigue life and, moreover, fatigue properties of 3D-printed PLA specimens. Indications of frequency-dependent fatigue parameters can already be observed from the direct results of the vibration-fatigue test (Figure 9), once the sample’s natural frequencies are considered. Utilizing the stress PSD from a valid numerical model, analysis with spectral method, and numerical minimization of the cost function provide a characteristic curve when comparing $T_{\mathrm{act}}$ to $T_{\mathrm{est}}$, shown in Figure 10. Dividing the trend curve of all samples (Figure 10) into sample sets within a given range of natural frequency, results in three linear segments. Performing the minimization of the cost function $Q(b,S_{f})$ yields different values of fatigue parameters *b* and $S_{f}$, whilst obtaining linear trends of $T_{\mathrm{act}}$ to $T_{\mathrm{est}}$. Generally, with the increase of loading frequency, the fatigue exponent *b* increases and the fatigue strength $S_{f}$ reduces.

Traditional fatigue testing methods result in significant temperature increase in polymer specimens at higher loading frequencies. This typically leads to reduced fatigue life due to thermal effects, as noted in previous studies [19,20]. However, using a novel vibration-based fatigue testing method [31], no relevant temperature increase occurred. Hence, the presented experimental results indicate that higher loading frequencies actually prolong the fatigue life of polymers within the observed stress load range as long as the temperature in the fatigue zone remains constant. This finding differs from earlier studies at lower loading frequencies (4–30 Hz) [17,18], and confirms the frequency dependence of fatigue parameters for 3D-printed PLA components.

The obtained results are significant for the design of 3D-printed polymer structures subjected to dynamic excitation within their natural frequency range. It is evident that fatigue parameters determined at low loading frequencies (4–30 Hz) cannot be extrapolated to higher frequencies, as this would result in overly conservative fatigue life estimates. The novel findings of this study demonstrate that the vibration-fatigue resilience of 3D-printed structures improves with increasing loading frequency. However, it is important to note that these results are specific to the tested material and the predefined manufacturing parameters. If there are variations in material or processing parameters, the vibration-fatigue testing procedure must be repeated to obtain reliable results. In addition to the broad range of polymer materials for 3D printing and processing parameters, the proposed methodology can also be utilized to evaluate the fatigue properties at different environmental conditions. This can be achieved by including a climatic chamber into the testing setup to control temperature and humidity.

## Figures and Tables

**Table 1:** Material properties and processing parameters.

Material Properties			Slicing Parameters
Material	PLA	Layer height	0.2 mm
Colorant	Blue	Infill	100% rectilinear
Supplier	Plastika Trček, Ljubljana, Slovenia	Raster angle	45°
Density	1333 kg/m^3^		
Filament diameter	1.75 mm		
Printing parameters			
Nozzle diameter		0.4 mm	
Nozzle temperature		220 °C	
Printing speed, external perimeter		25 mm/s	
Printing speed, internal perimeter		45 mm/s	
Printing speed, infill		80 mm/s	

**Table 2:** Quantity of tested samples for different PSD levels and weight lengths.

		Weight Length [mm]							
		9	12	15	18	21	24	27	30
$G_{\ddot{y}\ddot{y}}\left(\omega \right)$ level [g^2^/Hz]	0.10	4	4	4	4	2	2	1	2
	0.15	0	0	0	0	1	2	2	2

**Table 3:** Frequency dependence of fatigue parameters.

Frequency Range [Hz]	*b* [/]	$S_{f}$ [MPa]
250–350	−0.363	571.3
300–400	−0.167	56.93
450–700	−0.091	22.38
250–700	−0.148	48.60

## Data Availability

The original contributions presented in the study are included in the article, further inquiries can be directed to the corresponding author.

## References

[1] Gibson I., Rosen D., Stucker B., Khorasani M. (2000). Additive Manufacturing Technologies.

[2] Munghen D., Iacobellis V., Behdinan K. (2022). Incorporation of fiber Bragg grating sensors in additive manufactured Acrylonitrile butadiene styrene for strain monitoring during fatigue loading. Int. J. Fatigue.

[3] Gao S., Liu W., Zhang L., Gain A.K. (2020). A New Polymer-Based Mechanical Metamaterial with Tailorable Large Negative Poisson’s Ratios. Polymers.

[4] Liu T., Zhang M., Kang Y., Tian X., Ding J., Li D. (2023). Material extrusion 3D printing of polyether ether ketone in vacuum environment: Heat dissipation mechanism and performance. Addit. Manuf..

[5] Benamira M., Benhassine N., Ayad A., Dekhane A. (2023). Investigation of printing parameters effects on mechanical and failure properties of 3D printed PLA. Eng. Fail. Anal..

[6] Khosravani M.R., Berto F., Ayatollahi M.R., Reinicke T. (2022). Characterization of 3D-printed PLA parts with different raster orientations and printing speeds. Sci. Rep..

[7] Bakhtiari H., Aamir M., Tolouei-Rad M. (2023). Effect of 3D printing parameters on the fatigue properties of parts manufactured by fused filament fabrication: A review. Appl. Sci..

[8] Luo J., Luo Q., Zhang G., Li Q., Sun G. (2022). On strain rate and temperature dependent mechanical properties and constitutive models for additively manufactured polylactic acid (PLA) materials. Thin-Walled Struct..

[9] Afrose M.F., Masood S.H., Iovenitti P., Nikzad M., Sbarski I. (2016). Effects of part build orientations on fatigue behaviour of FDM-processed PLA material. Prog. Addit. Manuf..

[10] Rendas P., Imperadeiro A., Martins R.F., Soares B.A.R. (2024). High-Cycle Fatigue Behaviour of Polyetheretherketone (PEEK) Produced by Additive Manufacturing. Polymers.

[11] He F., Khan M. (2021). Effects of Printing Parameters on the Fatigue Behaviour of 3D-Printed ABS under Dynamic Thermo-Mechanical Loads. Polymers.

[12] Ziemian C., Ziemian R. (2020). Residual strength of additive manufactured ABS parts subjected to fatigue loading. Int. J. Fatigue.

[13] Travieso-Rodriguez J.A., Zandi M.D., Jerez-Mesa R., Lluma-Fuentes J. (2020). Fatigue behavior of PLA-wood composite manufactured by fused filament fabrication. J. Mater. Res. Technol..

[14] Terekhina S., Tarasova T., Egorov S., Skornyakov I., Guillaumat L., Hattali M. (2020). The effect of build orientation on both flexural quasi-static and fatigue behaviours of filament deposited PA6 polymer. Int. J. Fatigue.

[15] Li X., He J., Hu Z., Ye X., Wang S., Zhao Y., Wang B., Ou Y., Zhang J. (2022). High strength carbon-fiber reinforced polyamide 6 composites additively manufactured by screw-based extrusion. Compos. Sci. Technol..

[16] He Y., Huang W., Guo W., Li Y., Zhao S., Lin D. (2023). An Investigation of the Anisotropic Fatigue Properties of Laser Additively Manufactured Ti-6Al-4V under Vibration Loading. Materials.

[17] Ezeh O., Susmel L. (2019). Fatigue strength of additively manufactured polylactide (PLA): Effect of raster angle and non-zero mean stresses. Int. J. Fatigue.

[18] El Magri A., Vanaei S., Shirinbayan M., Vaudreuil S., Tcharkhtchi A. (2021). An Investigation to Study the Effect of Process Parameters on the Strength and Fatigue Behavior of 3D-Printed PLA-Graphene. Polymers.

[19] Shanmugam V., Das O., Babu K., Marimuthu U., Veerasimman A., Johnson D.J., Neisiany R.E., Hedenqvist M.S., Ramakrishna S., Berto F. (2021). Fatigue behaviour of FDM-3D printed polymers, polymeric composites and architected cellular materials. Int. J. Fatigue.

[20] Crawford R.J., Martin P.J. (2020). Plastics Engineering.

[21] Kuleyin H., Gümrük R., Çalışkan S. (2024). Fatigue behavior of polymethyl methacrylate/acrylonitrile butadiene styrene blends including blend composition, stress ratio, frequency, and holding pressure effects. Int. J. Fatigue.

[22] Nguyen H.T., Crittenden K., Weiss L., Bardaweel H. (2022). Experimental Modal Analysis and Characterization of Additively Manufactured Polymers. Polymers.

[23] Xue F., Robin G., Boudaoud H., Cruz Sanchez F.A., Daya E.M. (2022). General Methodology to Investigate the Effect of Process Parameters on the Vibration Properties of Structures Produced by Additive Manufacturing Using Fused Filament Fabrication. JOM.

[24] Medel F., Abad J., Esteban V. (2022). Stiffness and damping behavior of 3D printed specimens. Polym. Test..

[25] Huang Y.H., Lin C.Y. (2022). Measurement of Orthotropic Material Constants and Discussion on 3D Printing Parameters in Additive Manufacturing. Appl. Sci..

[26] Slavič J., Mršnik M., Česnik M., Javh J., Boltežar M. (2020). Vibration Fatigue by Spectral Methods.

[27] Palmieri M., Zucca G., Morettini G., Landi L., Cianetti F. (2022). Vibration Fatigue of FDM 3D Printed Structures: The Use of Frequency Domain Approach. Materials.

[28] Košir T., Slavič J. (2023). Manufacturing of single-process 3D-printed piezoelectric sensors with electromagnetic protection using thermoplastic material extrusion. Addit. Manuf..

[29] Humbert C., Barriol M., Varsavas S.D., Nicolay P., Brandstötter M. (2024). A Simple Method to Manufacture a Force Sensor Array Based on a Single-Material 3D-Printed Piezoresistive Foam and Metal Coating. Sensors.

[30] Palmić T.B., Slavič J. (2022). Single-process 3D-printed stacked dielectric actuator. Int. J. Mech. Sci..

[31] Česnik M., Slavič J., Boltežar M. (2023). Accelerated vibration-fatigue characterization for 3D-printed structures: Application to fused-filament-fabricated PLA samples. Int. J. Fatigue.

[32] Ewins D.J. (2000). Modal Testing: Theory, Practice and Application.

[33] Maia N.M.M., Silva J.M.M. (1997). Theoretical and Experimental Modal Analysis.

[34] Bendat J.S., Piersol A.G. (2010). Random Data: Analysis and Measurement Procedures.

[35] Bernasconi A., Kulin R.M. (2009). Effect of frequency upon fatigue strength of a short glass fiber reinforced polyamide 6: A superposition method based on cyclic creep parameters. Polym. Compos..

[36] Osti de Moraes D.V., Magnabosco R., Bolognesi Donato G.H., Prado Bettini S.H., Antunes M.C. (2015). Influence of loading frequency on the fatigue behaviour of coir fibre reinforced PP composite. Polym. Test..

[37] Naga S.A.R., El-Sayed T.A. (2024). Fatigue Failure in Polymeric Materials: Insights from Experimental Testing. J. Fail. Anal. Prev..

[38] Newland D.E. (1997). An Introduction to Random Vibrations, Spectral and Wavelet Analysis.

[39] Miles J.W. (1954). On Structural Fatigue under Random Loading. J. Aeronaut. Sci..

[40] Verboven P. (2002). Frequency-Domain System Identification for Modal Analysis. PhD Thesis.

[41] Zaletelj K., Bregar T., Gorjup D., Slavič J. (2020). pyEMA. https://github.com/ladisk/pyEMA.

[42] Capponi L., Česnik M., Slavič J., Cianetti F., Boltežar M. (2017). Non-stationarity index in vibration fatigue: Theoretical and experimental research. Int. J. Fatigue.

[43] Tang S., Wang X., Huang B., Yang D., Li L., He C., Xu B., Liu Y., Wang C., Wang Q. (2022). A Novel Ultrasonic Fatigue Test and Application in Bending Fatigue of TC4 Titanium Alloy. Materials.

[44] Dirlik T. (1985). Application of Computers in Fatigue Analysis. Ph.D. Thesis.

